# The illusion of safety: A report to the FDA on AI healthcare product approvals

**DOI:** 10.1371/journal.pdig.0000866

**Published:** 2025-06-05

**Authors:** Rawan Abulibdeh, Leo Anthony Celi, Ervin Sejdić

**Affiliations:** 1 Department of Electrical and Computer Engineering, University of Toronto, Toronto, Ontario, Canada; 2 Laboratory for Computational Physiology, Massachusetts Institute of Technology, Cambridge, Massachusetts, United States of America; 3 Division of Pulmonary, Critical Care and Sleep Medicine, Beth Israel Deaconess Medical Center, Boston, Massachusetts, United States of America; 4 Department of Biostatistics, Harvard T.H. Chan School of Public Health, Boston, Massachusetts, United States of America; 5 North York General Hospital, Toronto, Ontario, Canada; Washington University in Saint Louis, UNITED STATES OF AMERICA

## Abstract

Artificial intelligence is rapidly transforming healthcare, offering promising advancements in diagnosis, treatment, and patient outcomes. However, concerns regarding the regulatory oversight of artificial intelligence driven medical technologies have emerged, particularly with the U.S. Food and Drug Administration’s current approval processes. This paper critically examines the U.S. Food and Drug Administration’s regulatory framework for artificial intelligence powered healthcare products, highlighting gaps in safety evaluations, post-market surveillance, and ethical considerations. Artificial intelligence’s continuous learning capabilities introduce unique risks, as algorithms evolve beyond their initial validation, potentially leading to performance degradation and biased outcomes. Although the U.S. Food and Drug Administration has taken steps to address these challenges, such as artificial intelligence/machine learning-based software as a medical device action plan and proposed regulatory adjustments, significant weaknesses remain, particularly in real-time monitoring, transparency and bias mitigation. This paper argues for a more adaptive, community-engaged regulatory approach that mandates extensive post-market evaluations, requires artificial intelligence developers to disclose training data sources, and establishes enforceable standards for fairness, equity, and accountability. A patient-centered regulatory framework must also integrate diverse perspectives to ensure artificial intelligence technologies serve all populations equitably. By fostering an agile, transparent, and ethics-driven oversight system, the U.S. Food and Drug Administration can balance innovation with patient safety, ensuring that artificial intelligence-driven medical technologies enhance, rather than compromise, healthcare outcomes.

## Author Summary

Artificial intelligence is transforming U.S. healthcare, yet many AI-enabled tools are entering clinical use without rigorous evaluation or meaningful public scrutiny. Motivated by growing concerns over regulatory blind spots, especially during periods of deregulation and political pressure, we examined how the U.S. Food and Drug Administration (FDA) has reviewed and authorized AI medical devices to date. We reviewed publicly available FDA documentation and assessed the transparency and rigor of the evidence provided. Many tools lacked clear demonstration of clinical benefit or generalizability, and critical details such as testing procedures, validation cohorts, and bias mitigation strategies were often missing. We also identified inconsistencies in how the FDA categorizes and approves these technologies. Our findings raise urgent questions about the adequacy of current oversight and the pace at which AI technologies are integrated into clinical care. We offer practical policy recommendations to strengthen regulatory review and ensure that AI serves all patients, not just those easiest to include in development pipelines.

Artificial intelligence (AI) is poised to revolutionize every facet of medical practice, from streamlining notetaking and analyzing medical scans to enhancing diagnosis, treatment, and patient outcomes [1, 2]. In fact, the recent advancements of AI in healthcare have sparked active discussions about whether AI-powered systems might eventually replace human physicians. While it is unlikely that machines will replace human doctors in the foreseeable future, AI holds immense potential to assist physicians in making better clinical decisions and could even replace human judgment in certain specialized areas, such as radiology [3]. This transformative shift is fueled by the growing availability of healthcare data and rapid advancements in analytics techniques, offering the potential to reduce healthcare costs and mortality rates significantly [1, 4]. When guided by relevant clinical questions, powerful AI techniques can uncover clinically significant insights hidden within vast datasets, ultimately supporting and enhancing clinical decision-making processes [5, 6]. However, the US Food & Drugs Administration’s (FDA’s) recent wave of approvals for AI healthcare products raises a critical concern: Are these tools truly ready for deployment, or are they being prematurely validated, creating a false sense of security for clinicians and patients alike? [7–9]

The problems with the FDA’s approval process for AI in healthcare can be traced back to its early stages. Early FDA guidance classified some AI systems as “general wellness products," subject to loose regulation if deemed a low risk to users [3]. This foundational approach aimed to encourage innovation but failed to address the complexities of assessing safety and efficacy in AI systems [10, 11]. Today, although higher-risk AI-driven medical devices follow different regulatory pathways, these pathways may still not fully account for the unique risks and complexities of these technologies, raising concerns about the level of regulatory scrutiny they undergo [12]. Unlike static medical devices, the strength of artificial intelligence and machine learning (AI/ML)-based algorithms lies in their capacity for continuous learning, allowing them to evolve and improve based on real-world experience, often after the device or software has already been deployed for use. However, this evolution can introduce risks and biases that were not present and cannot be fully anticipated during pre-market evaluations, not only because of its dynamic nature but also because of AI’s sensitivity to contextual changes. These systems are termed adaptive algorithms by the FDA for which the traditional medical device regulatory framework was ill-equipped to monitor or mitigate [10, 11, 13]. Recognizing these gaps, the FDA introduced the AI/ML-based software as a medical device action plan in 2021, employing a “total product lifecycle" approach to provide robust oversight throughout the development and post-market phases–covering data quality, algorithm transparency, and robust change management [14, 15]. Building on this foundation, the FDA issued draft guidance in April 2023 for a predetermined change control plan designed to create a regulatory framework that accommodates the iterative and adaptive nature of AI/ML-enabled device software [16, 17]. This established a structured approach that lets developers address AI’s continuous evolution without repeatedly seeking new FDA approvals.

Despite these efforts, significant challenges remain in ensuring that these devices are consistently monitored for safe, effective, and equitable use as they evolve. While the FDA emphasizes post-market monitoring in its lifecycle approach, the implementation of robust, real-time monitoring mechanisms remains inconsistent and underdeveloped [18]. Continuous monitoring requires advanced infrastructure and significant resources, which are not yet fully integrated into the regulatory process and may even exceed the capacity of existing regulatory frameworks [16, 19–21]. The lack of continuous large-scale prospective evaluations of AI algorithms in diverse clinical settings can lead to unintended consequences which can even risk patient livelihood. Algorithms can change when exposed to new data, potentially leading to various problems, such as inaccurate predictions, reduced model effectiveness, and potential harm [22].

This inconsistency in post-market surveillance guidelines also leads to industry-wide variability, making it difficult to compare safety and performance across different AI/ML-enabled medical devices [18]. Moreover, transparency remains a significant issue—while post-market surveillance is a manufacturer’s responsibility, there is little visibility into how it is conducted or whether it effectively ensures long-term device safety. To improve oversight, post-market surveillance practices must be standardized to reduce variability in data collection and analysis, ensuring consistent monitoring of devices over time. Post-deployment evaluation should prioritize key healthcare metrics, including patient outcomes, health system performance, and workforce impact. This would provide a more accurate picture of an AI system’s real-world effectiveness and its influence on healthcare delivery, enabling the FDA to make informed, data-driven decisions that prioritize patient safety and system efficiency. Assessing the performance of a medical product’s model after deployment demands the same level of rigor as its premarket evaluation. Without proper monitoring, unregulated AI systems in practice could pose significant risks to patient safety.

There are also ethical implications that need to be considered for the employment of AI in healthcare including accountability, transparency, bias, fairness, permission, and privacy [22–24]. Issues of transparency are particularly important to address for deep learning algorithms [13]. Deploying devices that lack transparency or explainability and cannot be effectively evaluated by end users has the potential to exacerbate health disparities, particularly in the context of emerging clinical trials and real-world applications [7]. If these tools are making clinical decisions, it is essential to understand the reasoning and processes behind their decision-making to ensure transparency and trust. Embedding algorithmic transparency/explainability into regulatory frameworks is crucial for fostering accountability and providing greater reassurance to healthcare providers and patients [13]. Requiring manufacturers to provide transparent information about the functioning of AI/ML-enabled devices would allow the FDA to support a patient-centered approach, ensuring users have a comprehensive understanding of device applicability (i.e., their benefits, risks, and limitations).

In terms of bias and fairness, AI tools are only as good as the data they are trained on. When these datasets are biased or incomplete, the resulting systems perpetuate or even amplify biases reflecting patterns of social inequalities [22, 25–27]. Bias occurs when the validity of the outputs differs systemically across subpopulations [28]. This can disproportionately harm vulnerable groups like racial minorities, women, or socioeconomically disadvantaged individuals [29–31]. Such concerns are not specific to AI, but their rapid adoption can amplify these issues in the decision-making process and potentially exacerbate health inequalities, which can result in suboptimal or even harmful clinical decisions, especially if AI-generated recommendations are over-relied upon without critical evaluation [25, 32].

While recent initiatives by the FDA, such as the establishment of research programs aimed at understanding and measuring AI bias [33], indicate a growing recognition of broader concerns like fairness and equity, these efforts are still evolving and have yet to translate into enforceable regulations that explicitly address these concerns. For instance, the FDA’s non-binding transparency for machine learning-enabled medical devices guiding principles [34] outlines voluntary recommendations for interpretability and disclosure but stops short of providing enforceable standards. This gap in enforceable FDA regulations on ethical AI allows companies to rely on non-binding “soft law" guidelines, which, while promoting principles like fairness and transparency, often serve corporate interests [35–37]. These guidelines can be leveraged to downplay social issues or avoid stricter regulatory oversight [23]. As a result, systemic ethical challenges, including the need for robust evaluations of algorithmic performance across diverse populations, remain inadequately addressed. AI in health should be designed to inclusively account for age, sex, race, gender, ethnicity, sexual orientation, income, ability, and other protected characteristics [13]. Incentivizing AI developers to implement measures that minimize bias will allow regulatory authorities to support inclusiveness and equity. Without these measures, the FDA risks reinforcing existing inequities and missing opportunities to ensure that AI technologies in healthcare are not only safe and effective but also equitable and ethical.

A truly comprehensive regulatory framework for AI in healthcare must integrate ethical, technical, societal, and practical considerations to ensure responsible and effective oversight [13]. Establishment of a specialized regulatory body equipped with both legal authority and technical expertise to oversee AI development and deployment would address this need. This body must collaborate closely with universities, AI experts, and healthcare systems, leveraging their research capabilities and domain knowledge to enhance oversight. By fostering partnerships between academia, regulatory agencies, and health institutions we can create a proactive system that not only evaluates AI technologies throughout the life cycle but also prevents unsafe implementations before they reach deployment. Such a collaborative approach strengthens the regulatory landscape, ensuring that AI systems are rigorously tested, ethically aligned, and continuously monitored to protect public health.

The FDA’s regulatory framework would benefit from actively involving patients in the decision-making process, as they are the ultimate end-users of AI technologies in health, making their insights crucial to ensuring these products meet their needs and uphold safety standards. Their lived experiences and unique perspectives are essential for shaping regulations that address real-world needs, ensuring that AI tools are both user-friendly and impactful. While the FDA claims to incorporate public engagement into its regulatory framework, its current approach often feels performative, primarily soliciting feedback from individuals with the time, resources, and knowledge to participate. This process frequently excludes many patients and communities most vulnerable to harm from AI-driven healthcare innovations. True public engagement must be more expansive, inclusive, and intentional, ensuring that the perspectives of those most affected by these technologies — including through trusted patient and community advocates — have a voice in how these technologies are evaluated and implemented. Going beyond passive comment periods and actively seeking input from underrepresented communities would allow the FDA to craft regulations that reflect the diverse realities of patient care. Establishing mechanisms such as community advisory panels, participatory research initiatives, and partnerships with local health organizations can provide valuable insights that improve regulatory outcomes. By integrating these perspectives, AI governance can become more transparent, socially responsible, and attuned to real-world patient experiences [38]. Without deliberate inclusion of diverse populations, AI technologies risk reinforcing systemic biases, ultimately affecting the quality of care provided to marginalized groups [39]. To enable meaningful participation from all groups, it is essential to invest in improving AI literacy among patients, advocates, and community representatives. Providing stakeholders with a foundational understanding of AI technologies will empower them to engage more confidently and effectively in regulatory discussions and decision-making processes.

Beyond just addressing the challenges of bias, transparency, inclusivity, and post-market surveillance, the FDA needs to also address a fundamental regulatory challenge: determining which AI-driven health technologies warrant oversight and which may operate without stringent regulation. The rapid expansion of digital health technologies has blurred the lines between software as a medical device, non-device clinical decision support systems, and wellness applications. While software as a medical device is highly regulated, non-device clinical decision support systems and wellness applications often escape scrutiny despite their potential risks. According to the FDA’s updated guidance on non-device clinical decision support software, if a tool allows healthcare professionals to independently assess its recommendations, in some cases it may not be classified as a medical device and is therefore exempt from certain regulatory requirements [40]. However, this exemption raises concerns, as many clinicians may lack the time, resources, or expertise to fully evaluate these tools, potentially leading to reliance on unregulated software that may pose risks to patients [41]. Similarly, wellness applications and wearable devices, such as fitness trackers and health monitoring apps, are generally intended for non-medical use and often fall outside stringent regulatory oversight. While consumer health wearables offer benefits, concerns remain about their safety, reliability, potential overuse of healthcare services due to inaccurate readings, and the privacy and security of sensitive health data [42]. For example, some studies have linked wearables, particularly those detecting atrial fibrillation, to increased emergency department visits due to false positives [43]. These devices may not need the same stringent regulation as software as a medical device, but it is still crucial to study their impact thoroughly. Understanding and addressing these implications is essential to ensure their safe integration into healthcare.

As AI technologies grow more advanced, ensuring safety and validity through the FDA’s current regulatory methods is becoming increasingly impractical. This is especially true for continuously evolving AI systems that operate in high-stakes domains where errors or biases can have significant consequences. This necessitates an agile regulatory framework, one that is capable of continuous learning and adaptation. AI technology will continue to evolve in ways we may not yet anticipate. Current methods of continuous monitoring may be inadequate for future AI applications, and it is critical that regulations are designed to be adaptive rather than static. The FDA’s medical device regulatory framework, largely established in 1976, has remained relatively unchanged despite technological advancements. This rigidity highlights how overly conservative and stringent approaches can be counterproductive, particularly when addressing a dynamic and transformative technology like AI. A truly agile regulatory approach must be both reflective and reflexive, constantly evolving to support progress while ensuring that advancements do not deepen existing health disparities.

Only through an agile, feedback-driven, and community-engaged regulatory approach can we ensure that AI-driven medical innovations remain safe, equitable, and effective in improving patient care. To achieve this, we recommend a comprehensive framework that includes: (1) mandating extensive post-market monitoring and requiring updates to approvals if significant drift or performance degradation is detected, (2) ensuring AI developers disclose the composition of their training data and the methodologies behind their models for full transparency, (3) requiring evaluations of how AI tools perform across different demographic groups to mitigate biases, (4) involving a diverse range of stakeholders, including manufacturers, healthcare providers, patients, and regulatory bodies, in the development of AI regulations to ensure comprehensive and effective oversight, and (5) community-sourcing the regulation of health AI by actively including patients and their caregivers, especially those from historically underrepresented groups such as ethnic minorities, women and gender minorities, individuals from lower socioeconomic strata, people with disabilities, elderly individuals, and rural or underserved communities. The key to effective regulation is fostering a system that is constantly learning and consistently engaging with those most likely to be harmed by these technologies. Collectively, embedding these principles within FDA regulations will promote responsible and effective use of healthcare AI.

A practical implementation of these strategies can be achieved through structured partnerships with academic institutions. Leveraging the expertise of nursing, medical, engineering, and data science students offers healthcare systems access to a capable, cost-effective workforce for real-time monitoring and continuous evaluation of AI tools. Under faculty guidance, students can support data collection, model evaluation, and performance monitoring, contributing to the early detection of issues while gaining hands-on experience. This model also creates a valuable opportunity to integrate AI literacy and critical appraisal skills into clinical training, equipping future healthcare professionals to question AI outputs, recognize and address algorithmic errors and biases at the point of care, and serve as vital checkpoints for patient safety. While student turnover and variable skill levels present quality-control challenges, these can be mitigated through standardized onboarding procedures and close faculty mentorship to ensure consistent, high-quality assessments. Furthermore, clinician certification programs that are focused on AI ethics, bias detection, and system evaluation can reinforce critical engagement with AI tools. Tying these certifications to continuing education credits or licensure renewal requirements would incentivize clinicians to maintain up-to-date competencies and remain vigilant in their use of AI in practice.

Additional policy mechanisms can further promote the transparency and accountability of AI systems. Mandating the use of registries or open-access data repositories would allow developers, regulators, and researchers to monitor system performance collaboratively, expanding the evidence base for iterative improvements. By pooling real-world evidence, these repositories can help surface population-level biases or safety concerns that may only become apparent at scale. Independent auditing bodies—sanctioned by the FDA and specialized in AI—could conduct routine, impartial evaluations of AI tools both before and after approval. These evaluations would include public-facing summary reports detailing methods, datasets, performance outcomes, and any identified biases. This level of transparency can foster public trust and provide valuable feedback loops for both regulators and developers. Financial incentives, such as targeted grants or tax benefits for companies that meet high standards for data sharing and fairness, can further encourage equitable design. The FDA could offer expedited review pathways or tax credits for companies that proactively share source data, participate in open-access repositories, and incorporate diverse clinical validation practices. These incentives may also level the playing field for smaller, mission-driven startups competing against large tech firms.

To ensure regulatory alignment and efficiency, the FDA could collaborate with other federal agencies—such as the Office of the National Coordinator for Health Information Technology and the Centers for Medicare & Medicaid Services—to harmonize data standards, coverage policies, and enforcement strategies. Such interagency coordination would help streamline compliance for developers while ensuring more cohesive oversight. To foster community and patient engagement, the FDA could establish recurring forums where patient and community advocates, particularly from historically marginalized communities, review AI technologies, share user experiences, and offer input on inclusivity and usability. Partnering with nonprofit organizations and advocacy groups can also help build public understanding of how AI is used in healthcare, including its potential risks and benefits. Increasing baseline AI literacy will empower patients to better recognize algorithmic errors or biases and effectively communicate their concerns. Finally, the FDA should prepare for future readiness. Allocating federal research funding toward the development of regulatory frameworks for emerging technologies, such as autonomous clinical decision-making tools or large-scale predictive models used across health systems, can help anticipate challenges before they arise. This includes ensuring that guidelines for bias detection, transparency, and performance auditing can be updated rapidly as new use cases emerge. Regulatory agility is crucial in a field where rapid technological innovations can quickly outpace rigid oversight structures.

While no regulatory framework will ever be entirely foolproof, it is essential to safeguard both the technology and the people it impacts by building a system that is inclusive, adaptable, and forward-thinking. The target is constantly moving, for this reason, AI regulationsate the must focus on continuously measuring its effectiveness and adapting to ever-changing risks and threats: Is it appropriately balancing innovation and safety? Is it responsive to emerging challenges? By embracing this approach, the FDA can establish a system that not only protects public health but also fosters responsible and equitable innovation. While the FDA has made strides in improving AI/ML-based medical device oversight, the rapid pace of technological advancement necessitates continuous refinement of regulatory practices. In the world of AI-driven healthcare, stagnation is not just a potential regulatory failure, but a threat to patient safety and public trust.
